# Optimization of in-brace corrective force in adolescents with Lenke type 5 curve using finite element model

**DOI:** 10.1186/s13018-023-03857-8

**Published:** 2023-05-17

**Authors:** Kepeng Li, Jincheng Wu, Dongmei Yang, Hanpeng Xu, Wangqiang Wen, Haoxiang Xu, Guoju Ma, Ye Han

**Affiliations:** 1 Second Central Hospital of Baoding, Zhuozhou City, Hebei China; 2 Graduate School, Tianjin Medical University, Tianjin, China; 3 Southern Medical University, Guangzhou City, Guangdong China; 4 The First Affiliated Hospital of Hainan Medical University, Haikou City, Hainan China; 5 The Second People’s Hospital of Hefei, Anhui, China; 6 Affiliated Hospital of Hebei University, No. 214, Yuhua Road, Baoding City, Hebei China

**Keywords:** Lenke5 adolescent idiopathic scoliosis, Brace, Finite element analysis, Corrective forces, Pelvic tilt

## Abstract

**Background:**

Pelvic parameters have been taken into consideration for the evaluation of the outcomes of bracing in AIS. To discuss the stress required to correct the pelvic deformity related to Lenke5 adolescent idiopathic scoliosis (AIS) by finite element analysis, and provide a reference for the shaping of the pelvic region of the brace.

**Methods:**

An three-dimensional (3D) corrective force on the pelvic area was defined. Computed tomography images were used to reconstruct a 3D model of Lenke5 AIS. Computer-aided engineering software Abaqus was used to implement finite element analysis. By adjusting the magnitude and position of corrective forces, coronal pelvic coronal plane rotation (PCPR) and Cobb angle (CA) of lumbar curve in the coronal plane, horizontal pelvic axial plane rotation, and apical vertebra rotation (AVR) were minimized to achieve the best effect on the spine and pelvic deformity correction. The proposed corrective conditions were divided into three groups: (1) forces applied on *X*-axis; (2) forces applied both in the *X*- and *Y*-axis; and (3) forces applied along the *X*-, *Y*-, and *Z*-axis at the same time.

**Results:**

In three groups, CA correction reduced by 31.5%, 42.5%, and 59.8%, and the PCPR changed to 12°, 13°, and 1° from 6.5°, respectively. The best groups of correction forces should simultaneously locate on the sagittal, transverse, and coronal planes of the pelvis.

**Conclusions:**

For Lenke5 AIS, 3D correction forces can sufficiently reduce scoliosis and pelvic asymmetrical state. Force applied along the *Z*-axis is vital to correct the pelvic coronal pelvic tilt associated with Lenke5 AIS.

## Introduction

Adolescent idiopathic scoliosis (AIS) is a three-dimensional trunk deformity, including the spine and pelvis [1]. The global incidence of AIS ranges from 0.47 to 5.2% of adolescents aged 10–16 years old [2]. AIS can disturb the functional biomechanics of the body, limit lung volume, and reduce quality of life [3]. The brace is advocated as an effective method for treating mild to moderate AIS. Lenke5 type AIS is often combined with pelvic rotation and tilt deformity. Before brace treatment, it is necessary first to restore the pelvis to a neutral state and use the pelvis as the cornerstone of scoliosis correction [4].

The conservative treatment modalities of AIS range from physiotherapy and sports to brace therapy. Brace treatment is an established cornerstone of non-operative management and indicated for those patients with progressive curves (curves 25°–45°) [5]. The initial curve magnitude, type of the curve, and degrees of in-brace correction are prognostic risk factors for progression of the curve [6]. Pelvis is a necessary base of bracing to correct AIS. The pelvic region of traditional braces needs to be designed to be fully enclosed. During the manufacture of the brace, the pelvic area of the brace needs to be shaped symmetrically to the left and right sides. With the progress of orthopedic research on braces, it is hoped that a good scoliosis correction effect can be achieved with minimal body surface coverage, which can significantly improve the comfort of AIS patients, help improve the compliance of brace wearing, and further improve the effect of braces [7]. To achieve satisfactory scoliosis correction with the limited coverage area of the brace, precise design of the compression zone and opening area of the brace is required.

Previous studies on scoliosis brace correction first limited pelvic motion and analyzed the stresses exerted on the spine [8]. Mechanical studies of braces in the pelvic region have not been reported. In this study, finite element simulation biomechanics was used to analyze the orthopedic force in the pelvic region of the Lenke5 AIS, which laid a foundation for the design of better corrective braces.

## Methods

### Personalization of the AIS model

**Fig. 1:**
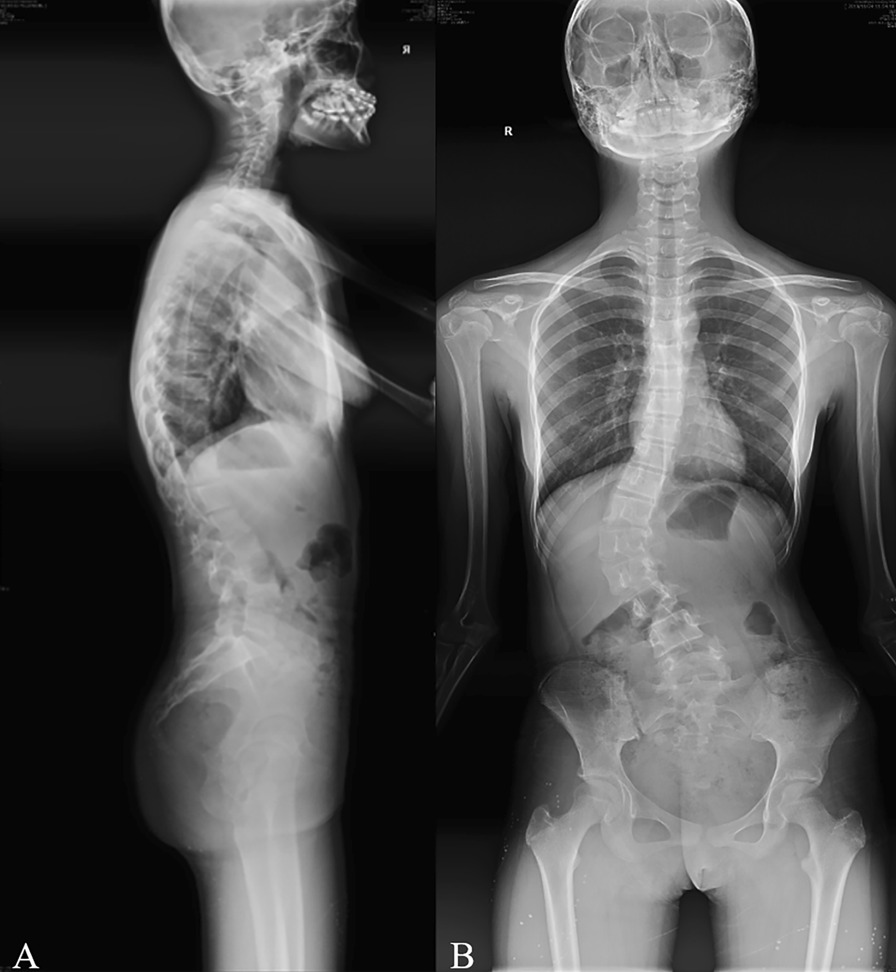
Biplane X-ray of the patient with Lenke5 AIS. **A** Lateral film. **B** Frontal film

The patient was Lenke5 AIS with right thoracolumbar scoliotic curvatures, with a Cobb angle of 40° (Fig. 1). The apex was located in the L1 vertebra. The scoliosis deformity at lumbar level is anatomically related to the pelvis in Lenke 5 AIS patients [9]. The patient was scanned by Philips Brilliance CT scanner from T1 to pelvis. The upper vertebra is the thoracic 11 vertebra. The lower vertebra is the lumbar 4 vertebra. The primary curve Cobb angle of lumbar curve in the coronal plane (CA) was 40°, pelvic coronal plane rotation (PCPR) 4°, pelvic incidence (PI) 43.9°, pelvic tilt (PT) 7.8°, and sacral slope (SS) 36.1°. Thoracolumbar kyphosis (TLK) was 27.3°, lumbar lordosis (LL) was 54.7°, and the lordotic apex of LL was located at the lumbar 4 vertebra.

The patient provided their written informed consent to participate in this study. All clinical investigations had been conducted according to the principles expressed in the Declaration of Helsinki. For CT scan and identifiable images used for the simulation study, this study has been approved by the Ethics Committee of Second Central Hospital of Baoding.

A 3D model of the Lenke5 AIS was created from CT images using medical image processing software (Mimics 20.0; Materialise, Belgium). The spine model was extracted based on the bone threshold, and the different regions were segmented using the area-growing tool. Finally, the 3D geometric models of the vertebrae, ribs, and pelvis were obtained for simulation. The model was imported into 3-Matic 12.0 software (Materialise Inc.) to perform wrapping, smoothing and Boolean operation. The redundant triangular surfaces were removed to generate more detailed 3D images, and the structures of facet joints, intervertebral disks and nucleus pulposus were initially constructed. Irregularly shaped disks have thresholds close to the muscle and are difficult to extract directly from CT images [8, 10]. The upper and lower surfaces of the vertebral bodies were taken out, and the corresponding cylinders were established. Obtain a 3D model of the intervertebral disk using Boolean operations in 3-Matics. The 3D spine model was optimized using reverse engineering software (Geomagic Wrap 2017; Geomagic, America) to facilitate the next biomechanical simulation. In Geomagic software, we performed denoising and smoothing to reconstruct the 3D model. Software (HyperMesh 2019; Altair; America) was used for meshing 3D models [11]. Vertebrae, intervertebral disks, and ribs were simulated using tetrahedral units and 7 ligaments with truss elements (ALL: anterior longitudinal ligament; PLL: posterior longitudinal ligament; LF: ligamentum flavum; CL: capsular ligament; ISL: interspinous ligament SSL: supraspinal ligament; ITL: Intertransverse ligament).

**Fig. 2:**
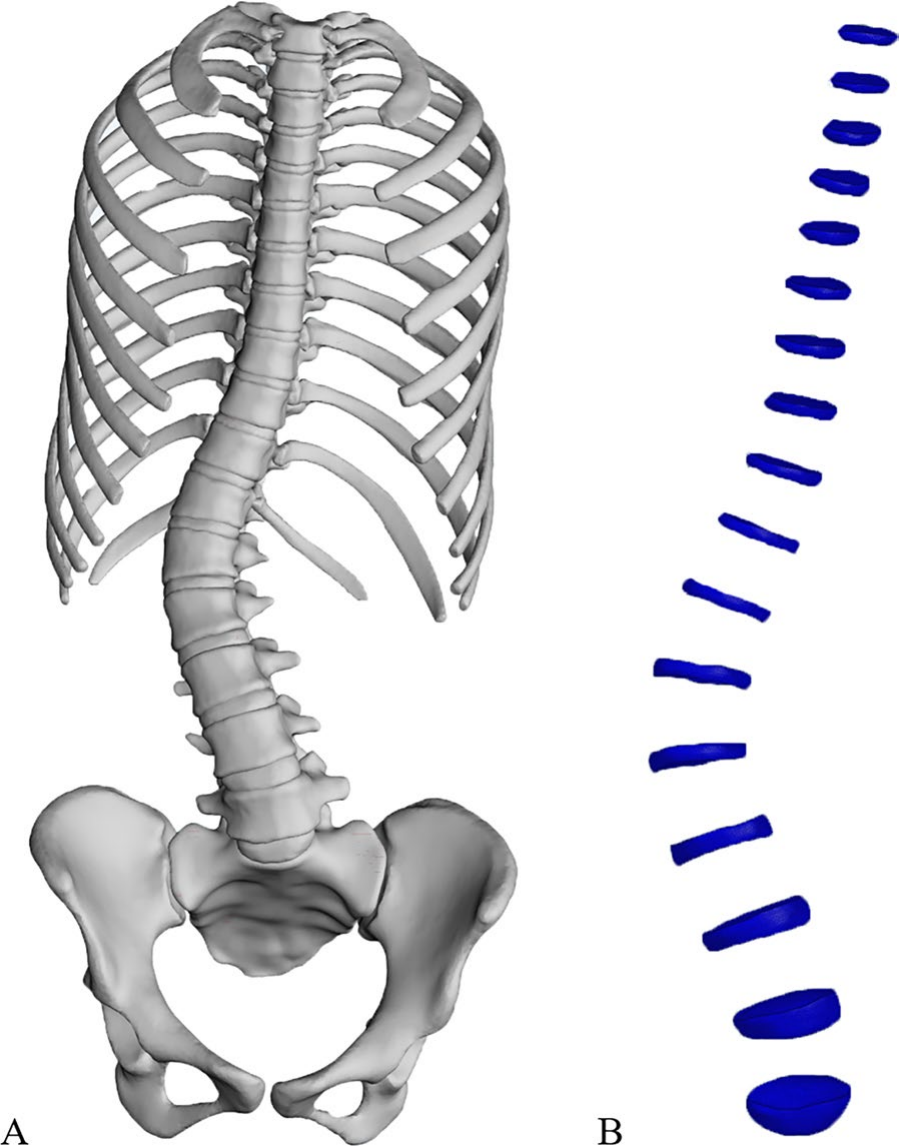
FEM of patient with Lenke5 AIS. **A** Complete finite element model of spine. **B** Finite element model of intervertebral disk

The AIS finite element model (FEM) is shown in Fig. 2. Based on the CT data, models of bones, intervertebral disks, and ligaments were established. Bones include the ribs, spine, and pelvis. The pelvis consists of the ilium, pubis, ischium, and sacrum. Ligaments were simulated by using a tension-only truss element [12]. Facet contact surfaces were defined as surface-to-surface contacts with a friction coefficient of 0.1 [13, 14]. In total, the spine model contained 550,177 elements and 147,221 nodes. Among them, 559,528 were C3D4 and 649 were T3D2. Mechanical properties of anatomical structures taken from published data (Table 1) [10, 15, 16].

**Table 1:** Mechanical properties of FEM anatomical structures

Component	Young’s Modulus (MPa)	Poisson ratio	Cross-sectional area (mm^2^)
Vertebral body	12,000	0.3	–
Rib	5000	0.3	–
disk	4.2	0.45	–
ALL	20	–	63.7
PLL	20	–	20
SSL	15	–	30
ITL	58.7	–	1.8
ISL	11.6	–	40
CL	32.9	–	30
LF	19.5	–	40

ALL, anterior longitudinal ligament; PLL, posterior longitudinal ligament; LF, ligamentum flavum; CL, capsular ligament; ISL, interspinous ligament SSL, supraspinal ligament; ITL, Intertransverse ligament

AIS models were processed and simulated in Computer-Aided Engineering software (Abaqus 2019; Dassault Systemes Simulia; France).

### Defining parameters

The coronal axis was *X*-axis, sagittal axis was *Y*-axis, and vertical axis was *Z*-axis in the coordinate system. The curvature curve was defined by connecting the center of each vertebral body to *Y*-axis. Curvature was determined based on the orientation of vertebral endplates on the *X*-axis [17, 18].

### Finite element model validation

The biomechanical characteristics of the FEM need to be consistent with the patient’s actual situation. The established model needs to be validated before performing simulated biomechanical analysis. The validity of the established FEM of scoliosis was verified. All T1–pelvis segments were used to simulate spatial positional changes of lateral bending under the external moment of force. As there were no experimental data on the biomechanics of AIS’s whole spine, we referred to the validation method of previous research [19, 20], reconstructed the geometry of the spine under different motion states, and compared the X-ray film with the FEM.

### Loading condition

**Fig. 3:**
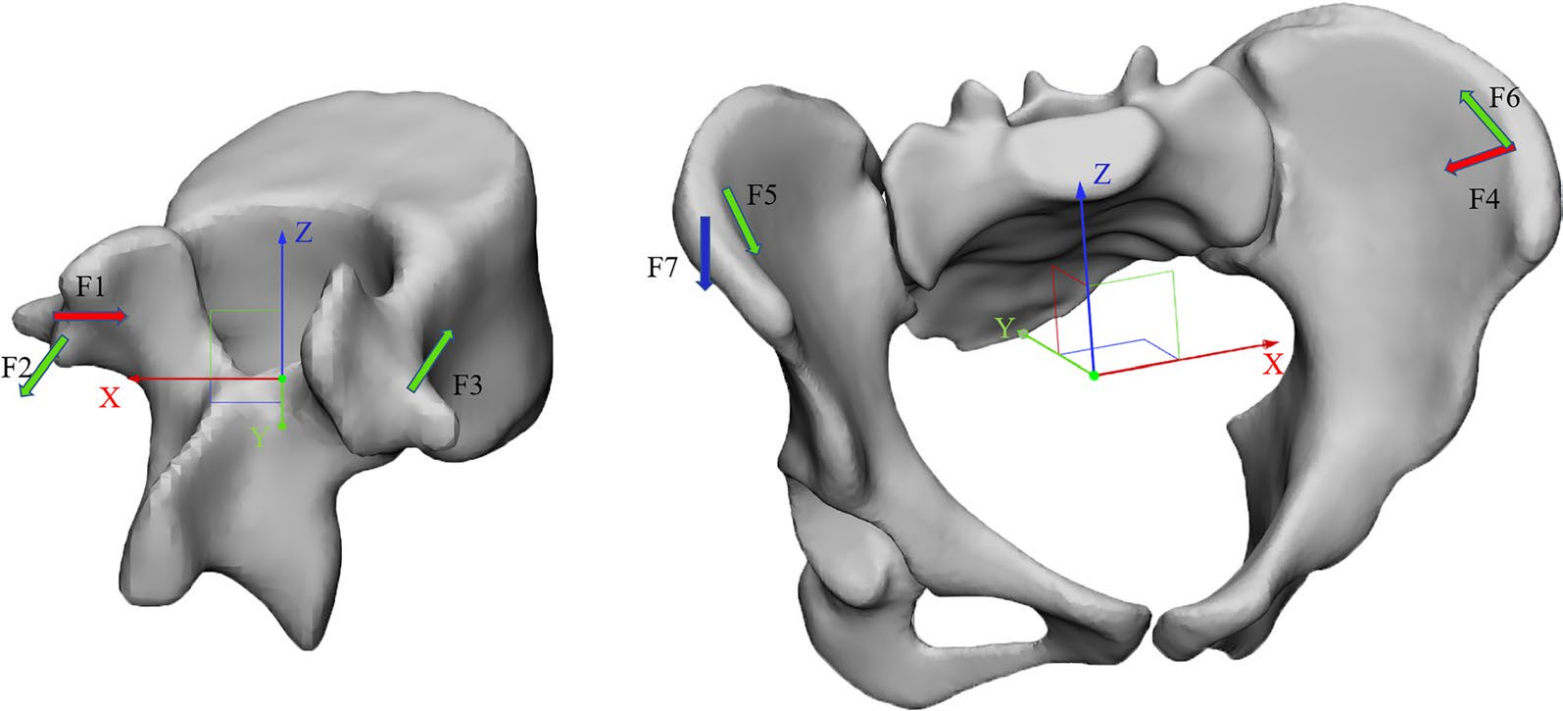
The schematic diagram of the direction in which the force was applied

According to the Hueter–Volkmann law, pressure on the epiphysis of the spine inhibits the growth of epiphysis. A three-point system was formed by force [21], and two counterforces were applied proximally and distally to the first one. The force applied should below 100N. The direction of the forces and counterforces was always from lateral to medial. Still, the pads (mainly lumbar and thoracic) providing the vector forces are oriented in an oblique plane rather than in a single frontal plane [22]. They will also provide the forces for derotation in the transversal plane. Three point force principle is applied to the scoliosis model to achieve correction effects in 3D space [23]. The applied force was divided into five groups (Fig. 3); (a) F1 was applied to L1 vertebrae along *X*-axis to correct coronal deviation; (b) Application of F2 and F3 in L1 vertebrae along *Y*-axis to correct vertebral rotation; (c) F4 was applied to Left ala of ilium along *X*-axis to provide distal counterforce of the three-point system; (d) Application of F5 and F6 in the right and left ala of ilium, respectively, along *Y*-axis to correct horizontal pelvic rotation; (e) F7 was applied to the right ala of ilium along *Z*-axis to correct coronal pelvic rotation [23–25].

Three sets of correction forces were applied to the model and compared. The first group included F1, F2, F3, and F4, which were used to the model at the same time; the second group included F1, F2, F3, F4, F5, and F6, which were applied to the model at the same time; the third group included F1, F2, F3, F4, F5, F6, and F7, which were applied to the model at the same time.

### The aim index was as

1. CA: The angle of two lines along with the superior endplate of the upper-end vertebra (T11) and the lower endplate of the lower end vertebra (L4) of the scoliosis model.
2. Apical vertebra rotation (AVR): As the vertex of this AIS model is located in L1, one node at the anterior edge of the L1 vertebra and one node at the tip of the L1 spinous process were taken, the angle of the connection between the two nodes and the sagittal plane.
3. TLK was defined as the angle between the upper endplate of the T10 vertebra and the lower endplate of the L2 vertebra.
4. LL: The angle between the superior endplate of L1 and the superior endplate of S1.
5. PCPR was defined as the angle between the line connecting the bilateral upper edge of the iliac wing and the horizontal line.
6. SS: The angle between the superior plate of S1 and a horizontal line.
7. Pelvic axial plane rotation (PAPR): One node was taken at the anterior edge of symphysis ossium pubis and one node at the dorsal center of the scoliosis model. The angle of the connection between the two nodes and the sagittal plane.
8. Coronary deviation: the distance between the vertical line passing through the center of T1 and the midline of S1 in the coronal plane.
9. Sagittal deviation: the distance between the vertical line passing through the center of T1 and the posterior upper horn of the S1 vertebra in the sagittal plane.

**Fig. 4:**
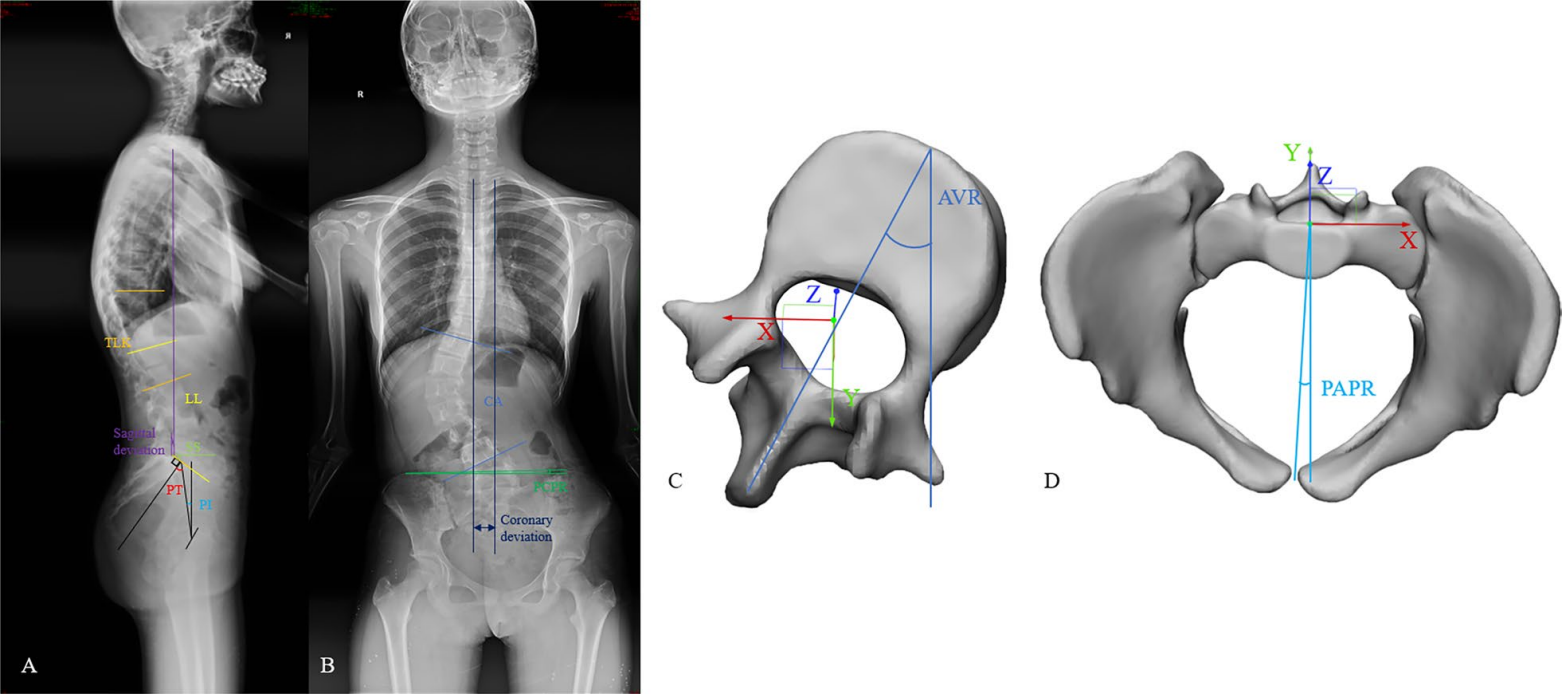
The parameter diagram of the spine. **A** Lateral film. **B** Frontal film **C** Upper view of the L1. **D** Upper view of pelvis

Figure 4 showed the parameter diagram of the spine. The optimal correction was considered when the spine and pelvis were symmetrical in horizontal and coronal planes. In actual clinical scoliosis and associated pelvic deformity correction process, the correction often cannot achieve the ideal state due to the complexity of muscles and other structures. Therefore, in treating scoliosis and associated pelvic deformity, the goal is to minimize coronal PCPR and CA, horizontal PAPR, and AVR simultaneously as much as possible [8, 26].

### Statistical analysis

In Finite Element Model Validation, IBM SPSS Statistics 25 was used to perform a paired *t* test on the data measured by FEM and X-ray, with *α* = 0.05.

## Results

**Fig. 5:**
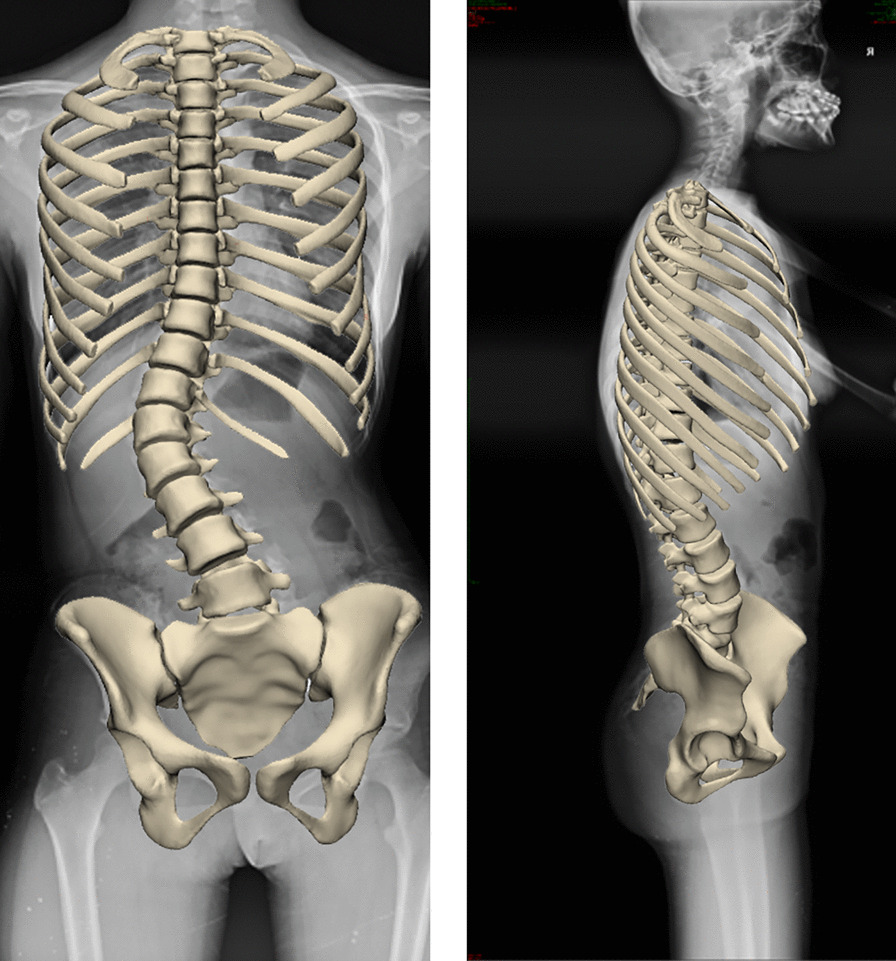
Morphological validation of the FEM

**Fig. 6:**
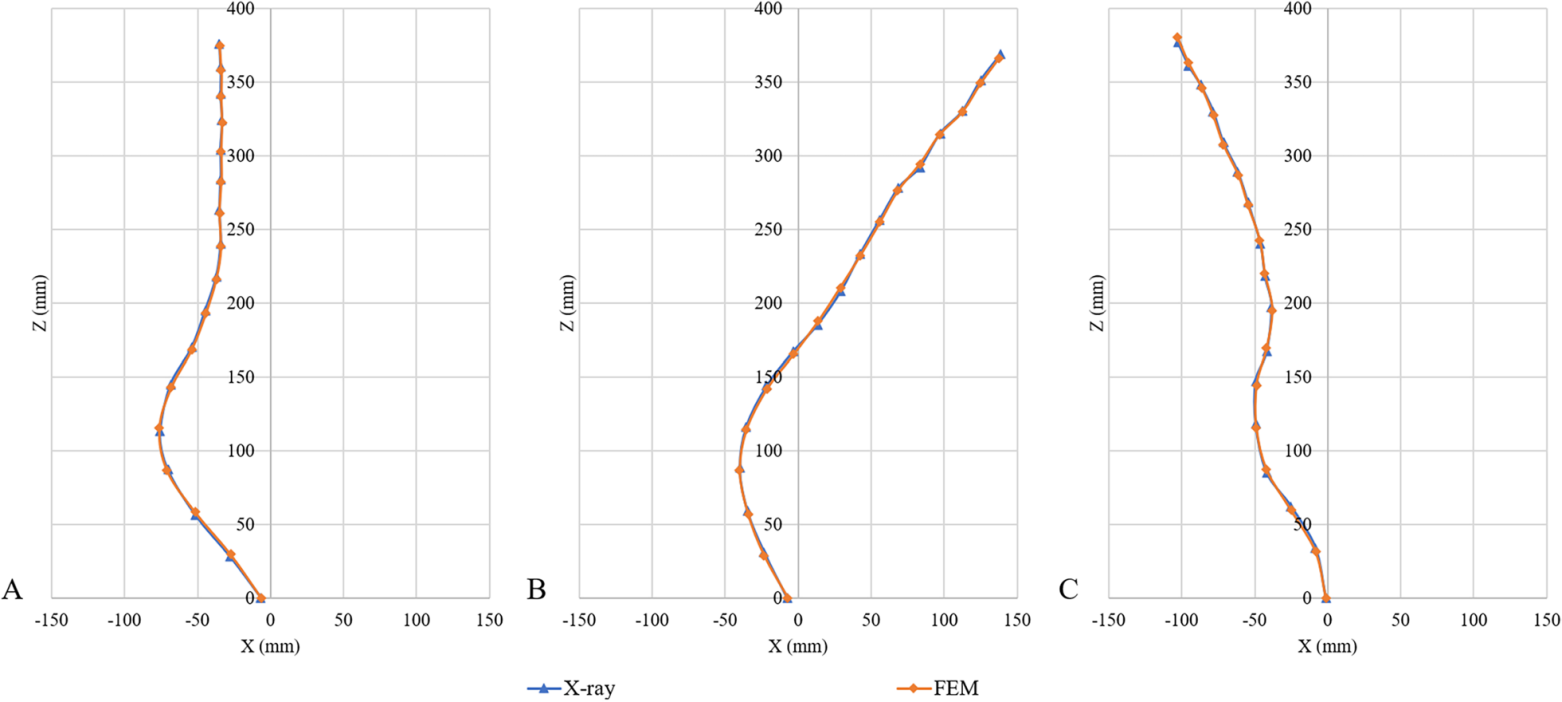
Comparison of the offset distance between the center of each vertebral body of the spine and the midline of the sacrum between the FEM and X-ray film. **A** Upright position **B** Left bending **C** Right bending

**Fig. 7:**
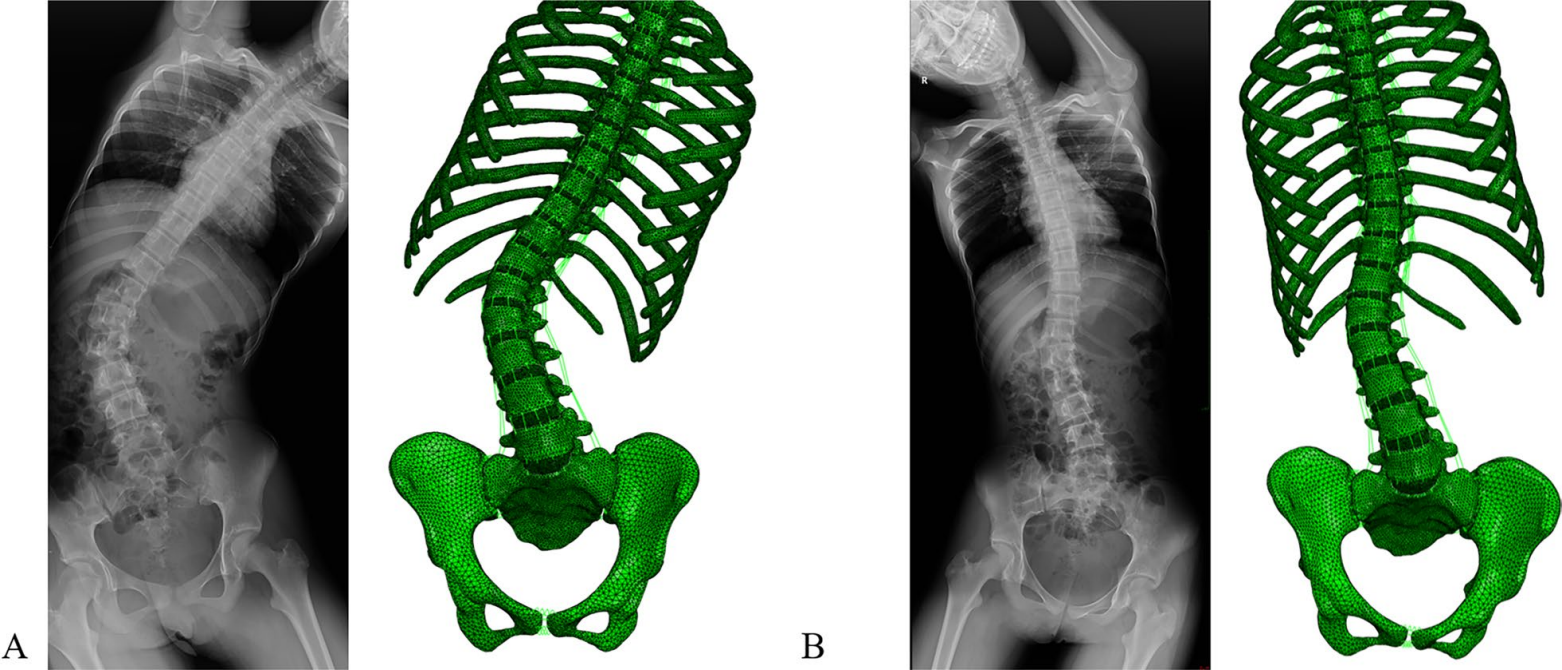
Morphological validation of lateral bending of the model. **A** Left bending **B** Right bending

The finite element model of Lenke5 AIS, including all thoracolumbosacral vertebrae, ribs, disks, ligaments, and facet join, was successfully established. The model was divided into 525,975 elements and 145,377 nodes; the model’s morphological validation showed basically the same shape as the X-line (Figs. 5 and 6). There is a good consistency between the central position of the vertebrae, the lateral bending angle of the sagittal and coronal plane, and the X-ray of their corresponding patients. The results of bending finite element simulation showed that the distance between T1 centrality to center sacral vertebrae line was agreeable with the measured data on the left and right Bending X-ray (Figs. 6 and 7, Table 2).

**Table2:** The offset distance between the model and X-ray film (cm)

Spinal segment	Frontal		Left bending		Right bending	
	X-ray	Model	X-ray	Model	X-ray	Model
T1	3.51	3.49	− 13.88	− 13.76	10.26	10.28
T2	3.41	3.38	− 12.51	− 12.49	9.53	9.55
T3	3.41	3.38	− 11.25	− 11.27	8.69	8.62
T4	3.32	3.30	− 9.73	− 9.69	7.85	7.83
T5	3.41	3.39	− 8.34	− 8.35	7.12	7.17
T6	3.41	3.39	− 6.88	− 6.82	6.18	6.13
T7	3.51	3.48	− 5.56	− 5.61	5.44	5.46
T8	3.41	3.40	− 4.24	− 4.27	4.61	4.66
T9	3.70	3.70	− 2.92	− 2.91	4.29	4.32
T10	4.46	4.45	− 1.33	− 1.38	3.87	3.81
T11	5.40	5.38	0.33	0.31	4.19	4.20
T12	6.83	6.81	2.18	2.13	4.92	4.88
L1	7.58	7.61	3.57	3.55	4.92	4.91
L2	7.02	7.10	3.99	4.01	4.19	4.20
L3	5.12	5.17	3.46	3.42	2.51	2.49
L4	2.75	2.74	2.33	2.33	0.84	0.82
L5	0.66	0.64	0.74	0.72	0.11	0.12
	*t*_1_ = 0.79		*t*_2_ = 0.22		*t*_3_ = 0.47	
	*p* = 0.44		*p* = 0.83		*p* = 0.65	

The simulation results showed that the optimal correction was achieved when correction forces were: F1 of 76N; F2 of 36N; F3 of 41N; F3 of 21N; F4 of 64N; F5 of 16N; F6 of 15N; F7 of 10.5N.

**Fig. 8:**
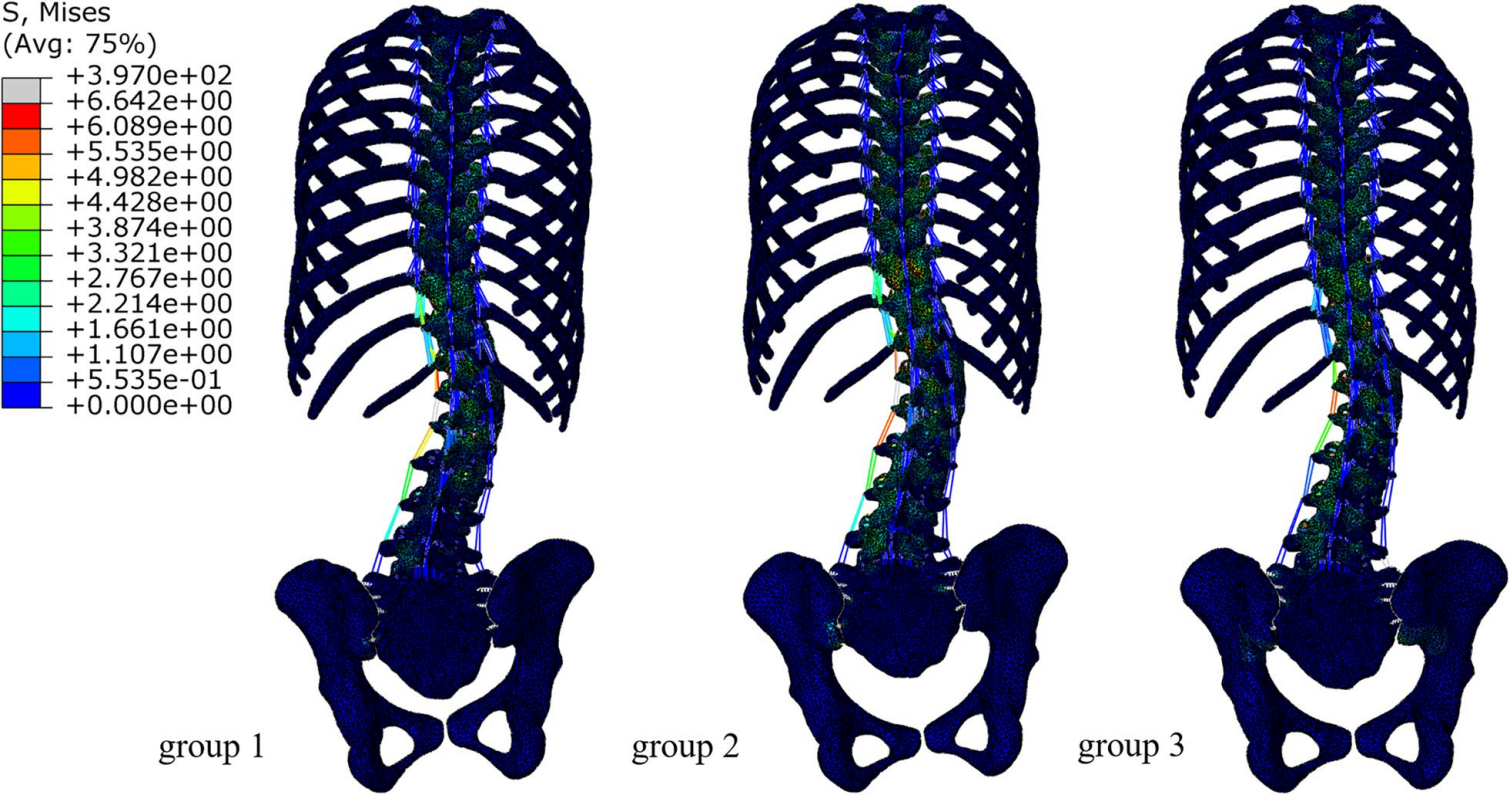
The stress diagram of the whole spine of Lenke5 AIS patient under three different correction methods

The CA correction of the first group was 31.5%, the force program of the first group showed increased PCPR and PT; CA correction of the second group was 42.5%, the force program of the second group showed increased PCPR; CA correction of the third group was 59.8%, the force program of the third group showed well 3D-balanced pelvis (Fig. 8, Table 3).

**Table 3:** Displacement of different orthopedic options for FEM

Index	Initial model	First group	Second group	Third group
CA (°)	40.0	27.4	23.0	16.1
AVR (°)	31.0	25.2	24.8	25.0
TLK (°)	27.3	14.7	13.6	15.2
LL (°)	54.7	45.0	42.3	37.2
PCPR (°)	6.5	12	13	1.0
SS (°)	39.4	30.5	25.9	34
PAPR (°)	15.0	10.6	4.2	1.8
Coronal deviation (cm)	3.4	0.8	0.1	0.1
Sagittal deviation (cm)	0.1	2.2	1.3	0.4

## Discussion

This is the first FEA study for the pelvic area design of braces. The pelvis plays a vital role in the occurrence and development of AIS. Guodong Wang et al. studied 52 cases of Lenke 5 AIS and found that PI influences sagittal spinal morphology in Lenke5 AIS [27]. Rob et al. measured the PI of thirty-seven female AIS patients and 44 non-scoliotic age-matched female controls, found Lenke type 5 patients showed a significantly higher PI than controls, propose a role of pelvic morphology and spinopelvic alignment in the pathogenesis of idiopathic scoliosis [1].

AIS is a three-dimensional deformity that occurs in the trunk. There were significant changes in the measurement of sagittal and vertebral body rotation after incorporating pelvic rotation factors in patients with AIS. Saba Pasha et al. retrospectively explore the effects of pelvic axial rotation on the coronal plane’s spinal balance in thirty-eight patients with AIS after posterior spinal instrumentation. They confirmed the existence of pelvic axial rotation in AIS patients and indicated that the pelvis would experience an active rebalancing in the transverse plane after spinal correction [28].

Moreover, lenke5 patients with preoperative pelvic rotation probably had a greater risk of coronal decompensation postoperatively [29]. Consistent with the literature, this research found that under the same stress conditions applied to the spine region, after applying pure *X*-axis stress, *X*-axis + *Y*-axis stress, and three-dimensional stress to the pelvic area, coronal CA correction reduced by 31.5%, 42.5%, 59.8%, respectively. It is suggested that pelvic rotation in the sagittal plane and transverse plane can affect the scoliosis correction effect in the coronal plane. This finding broadly supports the work of other studies in this area.

In this study, the basic orthopedic concept of the Lenke5 AIS model was based on the Cheneau brace. The three-point force orthopedic mechanism and the dual force anti-rotation mechanism were mainly used in the design of the brace. We try to maintain the patient’s physiological alignment on the sagittal plane and not perform excessive posterior pelvis rotation. The three-point force mechanism of the brace is mainly used to correct scoliosis on the coronal plane. Since the simulation boundary is located in T1, it can be considered that the head side of the scoliosis is relatively fixed, and there is no need to add a three-point force to the chest. The intermediate stress area is located on the lateral side of the apical vertebrae on the convex side of scoliosis. The distal stress area is located on the lateral side of the iliac crest on the concave side of scoliosis. The stress areas exerted by the three-point force can provide a larger force-bearing area, which is beneficial to the braces to exert the orthopedic effect on coronal scoliosis fully. Two pairs of dual forces can be applied when applying the brace against the rotation of the spine and pelvis in Lenke5 AIS, located in the apical region and the pelvis, respectively. The effect of maintaining the overall sagittal alignment of the patient can be achieved by antagonizing each other.

In the design concept of traditional braces in the past, the pelvis has always been regarded as the base of the spine [26]. The closed symmetrical pelvis design was first used to restore the pelvic rotation deformity during the brace design process. The rear of the pelvis is shaped parallel to the coronal plane. This design concept ignores the patient’s active avoidance mechanism. For patients with Lenke5 AIS, the open scoliosis brace is more comfortable than the previously closed brace, which is beneficial to improve patient compliance. The brace is designed to shape the pressure area in the stress area. The non-stress site is an open area, providing space for the patient to avoid and making full use of the patient’s active mechanism for orthopedics. For the design of the pelvic region of the brace, the anti-cross section rotation operation is mainly realized by the front and dual rear forces. The pressure area is designed in front of the anteriorly rotated hemipelvis and behind the posteriorly turned hemipelvis. In contrast, the open area is located posterior to the anteriorly rotated hemipelvis and anterior to the posteriorly rotated hemipelvis.

The finite element simulation orthopedic results of adding this dual force show that the rotational deformity of the pelvic cross section can be corrected from 15.0° before correction to 4.2°. However, only adding the orthopedic force in the *X*-axis direction in the pelvic region can only correct the rotational deformity of the pelvic cross section to 10.6°. When designing the pelvic area of the brace, it is suggested that it is necessary to apply the *Y*-axis stress according to the rotation state of the pelvis of the AIS patient.

Pelvic obliquity was frequently observed in patients with AIS, especially lumbar scoliosis [26]. Pelvic tilt can affect scoliosis and coronal alignment. Tomohiro Banno et al. revealed that ilium tilt was significantly associated with preoperative trunk imbalance and postoperative decompensation [30]. However, to our knowledge, few studies describe how to correct pelvic tilt during bracing,

The current study found that stress in the *Z*-axis direction is significant for correcting pelvic obliquity. When simulating orthopedics in the AIS model, the pelvic area needs to apply the *X*-axis orthopedic force as the distal stress of the three-point force system. The orthopedic force on the *Y*-axis improves the axial rotation of the pelvis. When only these two plane forces are applied. The pelvic obliquity increases from 6.5° to 13°, suggesting that stress in the *Z*-axis direction is also required to restore the balance of the pelvis in the three-dimensional plane. Since the force in the *Z*-axis direction needs to be applied at the iliac crest and the force area is small, only a small force (10.5N) was added in this study. One unanticipated finding was that pelvic obliquity was reduced from 13° to 1°. We can infer that a satisfactory orthopedic effect can be achieved only by applying a small stress in the *Z*-axis according to these data.

There is some limitation in this study. The role of muscle was not taken into account in this study. Since the description of the role of muscles in brace correction studies of scoliosis is controversial, whereas Wynarsky et al. affirmed that active muscle control plays an active role in brace correction and Odermatt et al. found significant changes in the electromyographic signal in the lumbar region of the patient’s spine [31, 32]. Similarly, Nie et al. affirmed that different parts of the body with different deformities exist to respond differently to the brace and that the patient’s muscles are able to react subconsciously to avoid the brace during the orthopedic process, thus playing an active role [10]. This active postural control of the muscular system is difficult to control quantitatively because the active control of the muscles arises under the passive control of the brace during correction and they are complementary to each other, amplifying the role of the brace in the correction process. And this feature limits the use of the model to analyze the role of muscles in treating patients with scoliosis. At the same time, in this study, the spinal model was simplified, and each component structure including the intervertebral disk and ligament was modeled as a linear element, and the material properties of each structure were assumed to be isotropic, consistent with the study of Mohammad et al. [11]. Also for the possible large displacements and deformations of the model, a convergence tolerance factor was chosen to reduce the computational cost while not significantly affecting the results of spine displacements (*Q*1). In future, we will further improve our experimental methods to make the design more reasonable and rigorous in order to improve our study.

## Conclusions

Lenke5 AIS is a three-dimensional deformity of the spine and pelvis. The three-dimensional deformation of the pelvic region is closely related to the patient’s overall alignment, coronal scoliosis angle, and vertebral body rotation in the apical vertebrae. Braces need to correct the three-dimensional rotation of the pelvis during orthopedic treatment. When correcting the three-dimensional rotation of the pelvis related to Lenke5 AIS, it is necessary to apply three-dimensional orthopedic force on the pelvis to improve the patient’s sagittal alignment, reduce the coronal tilt of the pelvis, and reduce the rotational deformity of the transverse plane. The pelvis on the concave side of scoliosis needs to bear the lateral force and the forward anti-rotation force applied at the rear. That is to say, the pressure zone of the brace needs to be designed behind the pelvis on the concave side of scoliosis; the pelvis on the convex side of scoliosis needs to be downward. Anti-tilt force and anti-rotation force to the rear, that is to say, it is necessary to design a pressure zone on the front and upper part of the pelvic iliac crest on the convex side of scoliosis. The rest of the pelvis is designed to be open, which utilizes the body’s avoidance mechanism for the pressure zone for orthopedics and reduces the coverage area of the brace to improve comfort and compliance.

## Data Availability

The datasets used and/or analyzed during the current study available from the corresponding author on reasonable request.

## Abbreviations

AIS: Adolescent idiopathic scoliosis
CA: Cobb angle of lumbar curve in the coronal plane
AVR: Apical vertebra rotation
TLK: The thoracolumbar kyphosis
LL: Lumbar lordosis
PCPR: Pelvic coronal plane rotation
PI: Pelvic incidence
PT: Pelvic tilt
SS: Sacral slope
PAPR: Pelvic axial plane rotation
FEM: Finite element model

## References

[1] Brink RC, Vavruch L, Schlösser T, Abul-Kasim K, Ohlin A, Tropp H, Castelein RM, Vrtovec T (2019). Three-dimensional pelvic incidence is much higher in (thoraco) lumbar scoliosis than in controls. Eur Spine J.

[2] Babaee T, Kamyab M, Ganjavian MS, Rouhani N, Khorramrouz A, Jarvis JG (2022). Coronal deformity angular ratio as a predictive factor for in-brace curve correction and long-term outcome of brace treatment in adolescents with idiopathic scoliosis. Spine Deform.

[3] Negrini S, Donzelli S, Aulisa AG, Czaprowski D, Schreiber S, de Mauroy JC, Diers H, Grivas TB, Knott P, Kotwicki T, Lebel A, Marti C, Maruyama T, O'Brien J, Price N, Parent E, Rigo M, Romano M, Stikeleather L, Wynne J, Zaina F (2018). 2016 SOSORT guidelines: orthopaedic and rehabilitation treatment of idiopathic scoliosis during growth. Scoliosis Spinal Disord.

[4] Khoshhal Y, Jalali M, Babaee T, Ghandhari H, Gum JL (2019). The effect of bracing on spinopelvic rotation and psychosocial parameters in adolescents with idiopathic scoliosis. Asian Spine J.

[5] Cheng JC, Castelein RM, Chu WC, Danielsson AJ, Dobbs MB, Grivas TB, Gurnett CA, Luk KD, Moreau A, Newton PO, Stokes IA, Weinstein SL, Burwell RG (2015). Adolescent idiopathic scoliosis. Nat Rev Dis Primers.

[6] Babaee T, Kamyab M, Ganjavian MS, Rouhani N, Jarvis J (2020). Success rate of brace treatment for juvenile-onset idiopathic scoliosis up to skeletal maturity. Int J Spine Surg.

[7] Li K, Miao J, Zhang J (2020). Pelvic rotation parameters related to in-brace correction in patients with idiopathic scoliosis. Eur J Med Res.

[8] Vergari C, Chen Z, Robichon L, Courtois I, Ebermeyer E, Vialle R, Langlais T, Pietton R, Skalli W (2021). Towards a predictive simulation of brace action in adolescent idiopathic scoliosis. Comput Methods Biomech Biomed Engin.

[9] Mac-Thiong JM, Labelle H, de Guise JA (2006). Comparison of sacropelvic morphology between normal adolescents and subjects with adolescent idiopathic scoliosis. Stud Health Technol Inform.

[10] Nie WZ, Ye M, Liu ZD, Wang CT (2009). The patient-specific brace design and biomechanical analysis of adolescent idiopathic scoliosis. J Biomech Eng.

[11] Karimi MT, Ebrahimi MH, Mohammadi A, McGarry A (2017). Evaluation of the influences of various force magnitudes and configurations on scoliotic curve correction using finite element analysis. Australas Phys Eng Sci Med.

[12] Li C, Zhou Y, Wang H, Liu J, Xiang L (2014). Treatment of unstable thoracolumbar fractures through short segment pedicle screw fixation techniques using pedicle fixation at the level of the fracture: a finite element analysis. PLoS ONE.

[13] Sengul E, Ozmen R, Yaman ME, Demir T (2021). Influence of posterior pedicle screw fixation at L4–L5 level on biomechanics of the lumbar spine with and without fusion: a finite element method. Biomed Eng.

[14] Clin J, Aubin CE, Parent S, Sangole A, Labelle H (2010). Comparison of the biomechanical 3D efficiency of different brace designs for the treatment of scoliosis using a finite element model. Eur Spine J.

[15] Wei W, Zhang T, Huang Z, Yang J (2022). Finite element analysis in brace treatment on adolescent idiopathic scoliosis. Med Biol Eng Comput.

[16] Vergari C, Ribes G, Aubert B, Adam C, Miladi L, Ilharreborde B, Abelin-Genevois K, Rouch P, Skalli W (2015). Evaluation of a patient-specific finite-element model to simulate conservative treatment in adolescent idiopathic scoliosis. Spine Deform.

[17] Périé D, Sales De Gauzy J, Hobatho MC (2002). Biomechanical evaluation of Cheneau–Toulouse–Munster brace in the treatment of scoliosis using optimisation approach and finite element method. Med Biol Eng Comput.

[18] Cheng FH, Shih SL, Chou WK, Liu CL, Sung WH, Chen CS (2010). Finite element analysis of the scoliotic spine under different loading conditions. Biomed Mater Eng.

[19] Périé D, Aubin CE, Petit Y, Labelle H, Dansereau J (2004). Personalized biomechanical simulations of orthotic treatment in idiopathic scoliosis. Clin Biomech (Bristol, Avon).

[20] Périé D, Aubin CE, Lacroix M, Lafon Y, Labelle H (2004). Biomechanical modelling of orthotic treatment of the scoliotic spine including a detailed representation of the brace-torso interface. Med Biol Eng Comput.

[21] Sculco PK, Windsor EN, Jerabek SA, Mayman DJ, Elbuluk A, Buckland AJ, Vigdorchik JM (2021). Preoperative spinopelvic hypermobility resolves following total hip arthroplasty. Bone Joint J..

[22] Shao X, Sui W, Deng Y, Yang J, Chen J, Yang J (2022). How to select the lowest instrumented vertebra in Lenke 5/6 adolescent idiopathic scoliosis patients with derotation technique. Eur Spine J.

[23] Anari JB, Tatad A, Cahill PJ, Flynn JM (2020). The impact of posterior spinal fusion (PSF) on coronal balance in adolescent idiopathic scoliosis (AIS): a new classification and trends in the postoperative period. J Pediatr Orthop.

[24] Bylski-Austrow DI, Dolan LA (2021). Spine growth modulation with titanium implant: comparisons to observation and bracing in early adolescent idiopathic scoliosis. Stud Health Technol Inform.

[25] Zuckerman SL, Cerpa M, Sardar ZM, Lenke LG (2021). Don't forget the pelvis: accounting for pelvic rotation in the preoperative assessment of adolescent idiopathic scoliosis. J Spine Surg.

[26] Saeedi M, Kamyab M, Babaee T, Behtash H, Ganjavian MS (2020). The effects of bracing on sagittal spinopelvic parameters and Cobb angle in adolescents with idiopathic scoliosis: a before-after clinical study. Turk J Phys Med Rehabil.

[27] Wang G, Li Y, Liu P, Sun J (2021). Pelvic incidence correlates to sagittal spinal morphology in lenke 5 adolescent idiopathic scoliosis and influences the proximal junctional kyphosis rate after correction surgery. Eur Spine J.

[28] Pasha S, Sankar WN, Castelein RM (2019). The link between the 3D spino-pelvic alignment and vertebral body morphology in adolescent idiopathic scoliosis. Spine Deform.

[29] Kato S, Zeller RD, Magana SP, Ganau M, Oshima Y, Tanaka S, Lewis SJ (2020). Postoperative distal coronal decompensation after fusion to L3 for adolescent idiopathic scoliosis is affected by sagittal pelvic parameters. Spine (Phila Pa 1976).

[30] Banno T, Yamato Y, Oba H, Ohba T, Hasegawa T, Yoshida G, Arima H, Oe S, Mihara Y, Ushirozako H, Takahashi J, Haro H, Matsuyama Y (2021). Preoperative pelvic obliquity: possible relation to postoperative coronal decompensation in thoracolumbar/lumbar adolescent idiopathic scoliosis. J Neurosurg Spine.

[31] Wynarsky GT, Schultz AB (1989). Trunk muscle activities in braced scoliosis patients. Spine (Pjila Pa 1976).

[32] Odermatt D, Mathieu PA, Beauséjour M, Labelle H, Aubin CE (2003). Electromyography of scoliotic patients treated with a brace. J Orthop Res.

